# Characterisation of an influenza B virus-derived peptide presented by HLA-B*18:01

**DOI:** 10.1042/BCJ20240739

**Published:** 2025-07-16

**Authors:** Lawton D. Murdolo, Samuel Liwei Leong, Janesha C. Maddumage, Nicole A. Mifsud, Demetra S.M. Chatzileontiadou, Emma J. Grant, Stephanie Gras

**Affiliations:** 1 Infection and Immunity Program, La Trobe Institute for Molecular Science (LIMS), Victoria, Bundoora VIC, Australia; 2 Department of Biochemistry and Chemistry, La Trobe University, Victoria, Bundoora VIC, Australia; 3 Immunity Program, Department of Biochemistry and Molecular Biology, Biomedicine Discovery Institute, Monash University, Clayton, VIC, Australia; 4 Department of Biochemistry and Molecular Biology, Monash University, Clayton, VIC, Australia

**Keywords:** CD8^+^T cells, HLA-B*18:01, influenza B virus, molecular dynamics, X-ray crystallography

## Abstract

The influenza B virus (IBV) can pose a significant threat to global health. Despite this, IBV is understudied compared with influenza A virus (IAV). CD8^+^ T cells have proven highly effective in reducing influenza disease severity. In addition, pre-existing immune responses towards IAV epitopes may provide protection against homologous IBV-derived peptides due to T cell cross-reactivity. To exploit the advantages of T cells for future vaccine developments, a better understanding of the IBV-derived peptide presentation by the highly polymorphic human leukocyte antigen (HLA) is required. We previously determined that the IAV-derived PB1_177-A_ peptide was presented by the HLA-B*18:01 molecule and induced CD8^+^ T cell responses. Here, we assessed the PB1_177-A_ IBV homologue (PB1_177-B_). Intracellular cytokine staining assays showed that PB1_177-B_ was unable to activate CD8^+^ T cells from several HLA-B*18:01+ samples tested. We determined that the IAVand IBV-derived PB1_177_ adopted different stability and conformation in the cleft of HLA-B*18:01. Molecular dynamics analysis on the crystal structure showed that PB1_177-B_ had a central flexible region with a large hydrophobic patch formed by two phenylalanine residues, not present in PB1_177-A_. The flexibility and the lower stability of the HLA-B*18:01-PB1_177-B_ complex may hinder CD8^+^ T cell receptor binding, underpinning the lack of CD8^+^ T cell activation observed. This provides additional insights into the differences between IAVand IBV-specific CD8^+^ T cell responses.

## Introduction

Co-circulating influenza viruses A and B (IAV and IBV, respectively) have devastating social and economic consequences around the world, with epidemics leading to an estimated 3–5 million cases globally with an estimated mortality rate between ~290,000 and ~650,000 individuals [1]. While current vaccines induce the production of antibodies through the activation of B cells, antigenic drift results in viral mutation, in particular within the surface viral glycoproteins haemagglutinin (HA) and neuraminidase (NA) [2]. Many of these point mutations essentially restrict the binding of neutralising antibodies (nAbs) to the virus, resulting in the inability of nAbs to bind the virus, thus leading to viral escape [3]. Therefore, influenza vaccines must be reformulated annually to closely match the circulating strains, costing up to US$ 68 billion [4]. These vaccines also rely on the World Health Organisation (WHO) to predict future circulating strains, which can result in yearly variable vaccine efficacy [5].

While protection induced by the seasonal influenza vaccine is heavily reliant on humoral immunity, cellular immunity through activation of CD8^+^ T cells could represent future targets for vaccine development. CD8^+^ T cells kill infected cells through the secretion of cytolytic molecules and cytokines and have been shown to provide a strong rapid response to IBV infection, in the absence of nAbs [6]. This is due to CD8^+^ T cells being able to recognise conserved regions of the virus including internal proteins, which are typically less susceptible to mutation as they are necessary for viral replication. CD8^+^ T cell activation is driven by the recognition of human leukocyte antigen class I molecules (HLA-I) on the surface of infected cells. Viral peptides are loaded onto the HLA within the cell, and this step required a stable peptide HLA (pHLA) complex [7]. The stable pHLA complexes are then transported to the surface where they can be displayed to CD8^+^ T cells, recognised via their T cell receptor (TCR). However, HLA-I molecules are highly polymorphic with over 28,000 allomorphs currently described [8]. Each HLA allomorph can bind a unique peptide sequence termed ‘peptide-binding motif’, which requires specific residues at position two (P2) and at the last position (PΩ), both primary peptide anchor residues [9].

To determine candidate epitopes for vaccines that can stimulate CD8^+^ T cells, a better understanding of peptide presentation from different HLA-I is required. However, characterisation of HLA-specific influenza-derived peptides has been mainly focused on IAV [10], potentially due to higher pandemic potential and unique zoonotic reservoirs [11]. On the other hand, IBV infections can be responsible for severe influenza diseases, especially in children, which include high rates of hospitalisation [12] and mortality [13]. However, the knowledge about HLA-I presentation of IBV-derived peptides or their activation of CD8^+^ T cells is limited.

Our work focuses on HLA-B*18:01 that is expressed in around 2.3% of the world’s population, spanning various continents [14]. Only structures of HLA-B*18:01 presenting IAV-derived peptides are available [15,16] but not IBV peptide. We previously showed that the IAV-derived peptide PB1_177-A_ was immunogenic in CD8^+^ T cells from HLA-B*18:01^+^ samples [16] and decided to investigate its homologous IBV peptide, termed PB1_177-B_. Despite the PB1_177-A_ and PB1_177-B_ only sharing a conserved primary anchor P2-Glu residue, both peptides have hydrophobic residues at P3 and P7 previously shown to help with the stability of the peptide in the HLA-B*18:01 binding cleft [16], suggesting that PB1_177-_B might form a stable complex with HLA-B*18:01. In addition, the PB1_177-B_ has a large side chain residue at PΩ (P9-Lys), and even if lysine is not a favoured residue for this primary anchor [17], it might enable the peptide to bind the HLA-B*18:01 molecule. Our study shows that, indeed, the PB1_177-B_ could bind to HLA-B*18:01 but failed to activate CD8^+^ T cells in several HLA-B*18:01^+^ samples tested. The crystal structure of the HLA-B*18:01-PB1_177-B_ complex showed a flexible central peptide region made up of hydrophobic residues, which was not present in the PB1_177-A_ epitope. This provides a potential structural basis for the lack of immunogenicity observed for PB1_177-B_ despite binding to HLA-B*18:01.

## Results

### The PB1_177-B_ peptide does not activate CD8^+^ T cells while PB1_177-A_ is immunogenic

We previously characterised an IAV-derived peptide, restricted to HLA-B*18:01 [16] from the IAV polymerase basic protein (PB1_177-A_). This peptide was strongly immunogenic in ~40% of the samples tested (*n* = 2/5) and the PB1_177-A_-specific T cells showed a polyfunctional profile. Sequence alignment of IAV (A/Darwin/9/2021, H3N2) and IBV (B/Wisconsin/22/2024, B/Victoria lineage) revealed limited similarity between the homologous peptides (Table 1). Despite the limited homology of the PB1_177-A_ and PB1_177-B_ peptides, both conserved the critical glutamic acid residue at position 2 (P2-E), important to bind HLA-B*18:01 molecule [19].

**Table 1:** Peptide sequence and thermal stability of HLA-B*18:01 presenting influenza-derived peptides.

Peptide name	Strains	Peptide sequence	*T*_m_ (°C) ± S.E.M.
PB1_177-B_	B/Wisconsin/22/2024 (B/Victoria lineage)	PEMTFFSVK	48.92 ± 1.25
PB1_177-A_ [16]	A/Darwin/9/2021 (H3N2) A/Victoria/2570/2019 (H1N1)	EEIEITTHF	64.55 ± 0.99
M1_5_ [16]	A/X‐31 /H3N2 A/Darwin/9/2021 (H3N2) A/H3N2/Wisconsin/67/2005 (H3N2) A/Victoria/2570/2019 (H1N1)	TEVETYVL	62.51 ± 0.04

The PB1 protein sequences from the influenza virus strains above were downloaded from NCBI Protein for B/Wisconsin/22/2024 (accession number: XBP28071) and A/Michigan/06/2009 (H1N1) (accession number: ACS72610) and were aligned using Clustal Omega Multiple Sequence Alignment Tool (18) to determine the homologous IBV-derived peptide. Tm represents the temperature at which 50% of the protein is unfolded and reported as the mean with standard error of the mean (S.E.M.) from two independent experiments performed in duplicate at two concentrations.

**Figure 1:**
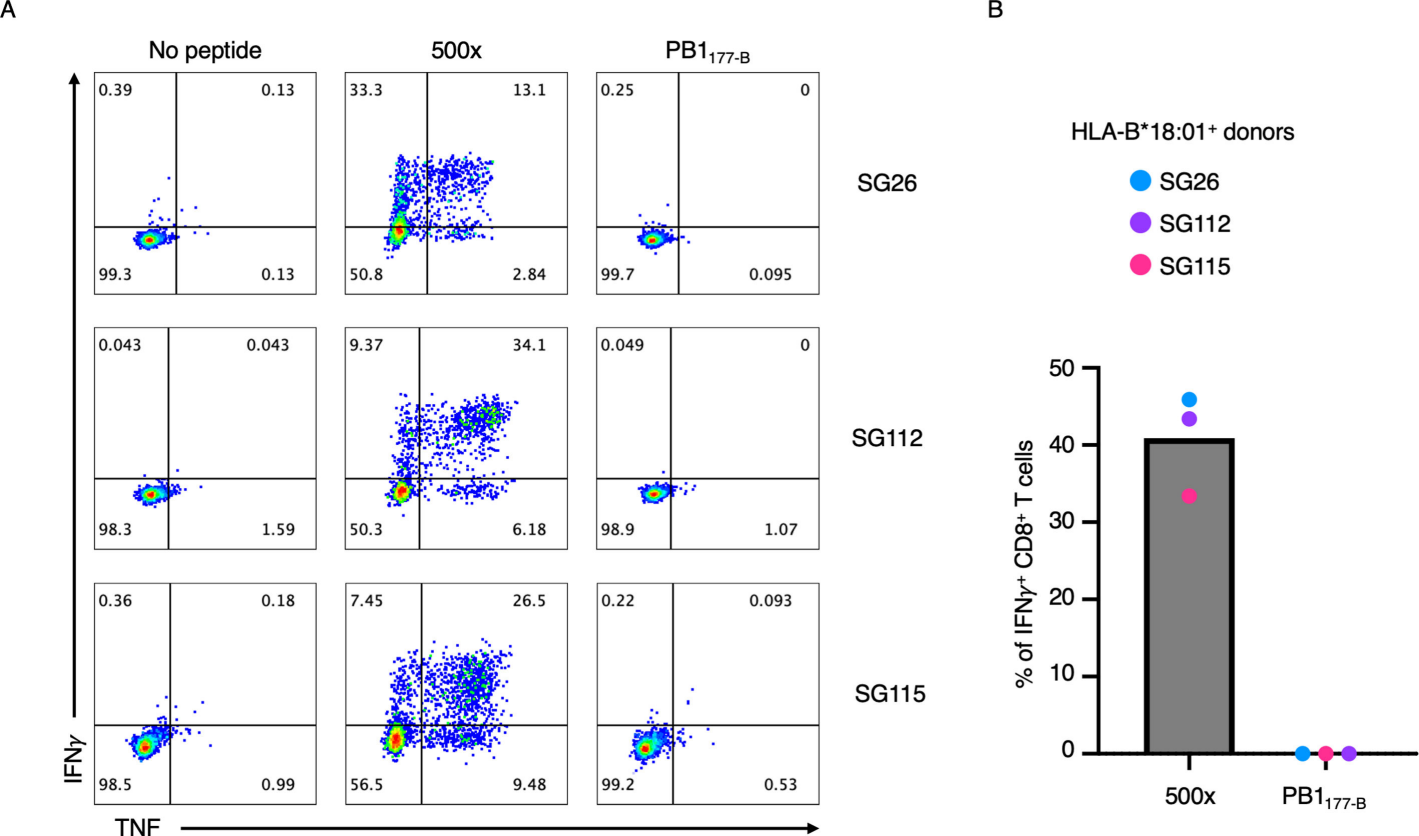
FACS plot and bar graph of cytokine production PBMCs isolated from HLA-B*18:01^+^ donors SG26, SG112 and SG115 were stimulated with PB1_177-B_ at 10 µM for a period of 10 days (*n* = 3). Post CD8^+^ T cell expansion, an ICS assay was completed by restimulating each PB1_177-B_-specific CD8^+^ T cell line with PB1_177-B_ at 10 µM. A positive control 500X condition and nil peptide negative control was completed for each CD8^+^ T cell line. (**A**) FACS plots for SG26, SG112 and SG115 with nil peptide, 500X (positive control) and peptide restimulation conditions are shown. (**B**) A bar graph displaying the percentage of IFNγ^+^ CD8^+^ T cells detected for each peptide via flow cytometry. Each dot represents a donor’s CD8^+^ T cell response, while the bars illustrate the average for each peptide (blue; SG26, purple; SG112 and pink; SG115).

We first determined whether the IBV homologous peptide, PB1_177-B_, could activate CD8^+^ T cells. Using PB1_177-B_ pulsed peripheral blood mononuclear cells (PBMCs) derived from three HLA-B*18:01^+^ donors, we generated CD8^+^ T cell lines. The level of CD8^+^ T cell activation was determined by the production of both IFNγ and TNF in an ICS assay (Figure 1, supplementary figure 1). While CD8^+^ T cell activation was present in the positive control (500 x), no IFNγ or TNF were produced upon restimulation with the PB1_177-B_ (Figure 1). The lack of CD8^+^ T cell activation was observed in all three samples tested, even in cells from the SG115 donor, which showed T cell activation towards the IAV-derived PB1_177-A_ in our previous study [16].

Therefore, in the samples tested, CD8^+^ T cell activation upon presentation of the PB1_177-B_ was absent. Given the sequence differences of the PB1_177-B_ compared with PB1_177-A_, and the presence of a C-terminal lysine, which is an unusual PΩ residue for HLA-B*18:01, it is possible that the PB1_177-B_ is not presented by the HLA-B*18:01 molecule.

### The PB1_177-B_ peptide central region is flexible in the cleft of HLA-B*18:01

**Figure 2:**
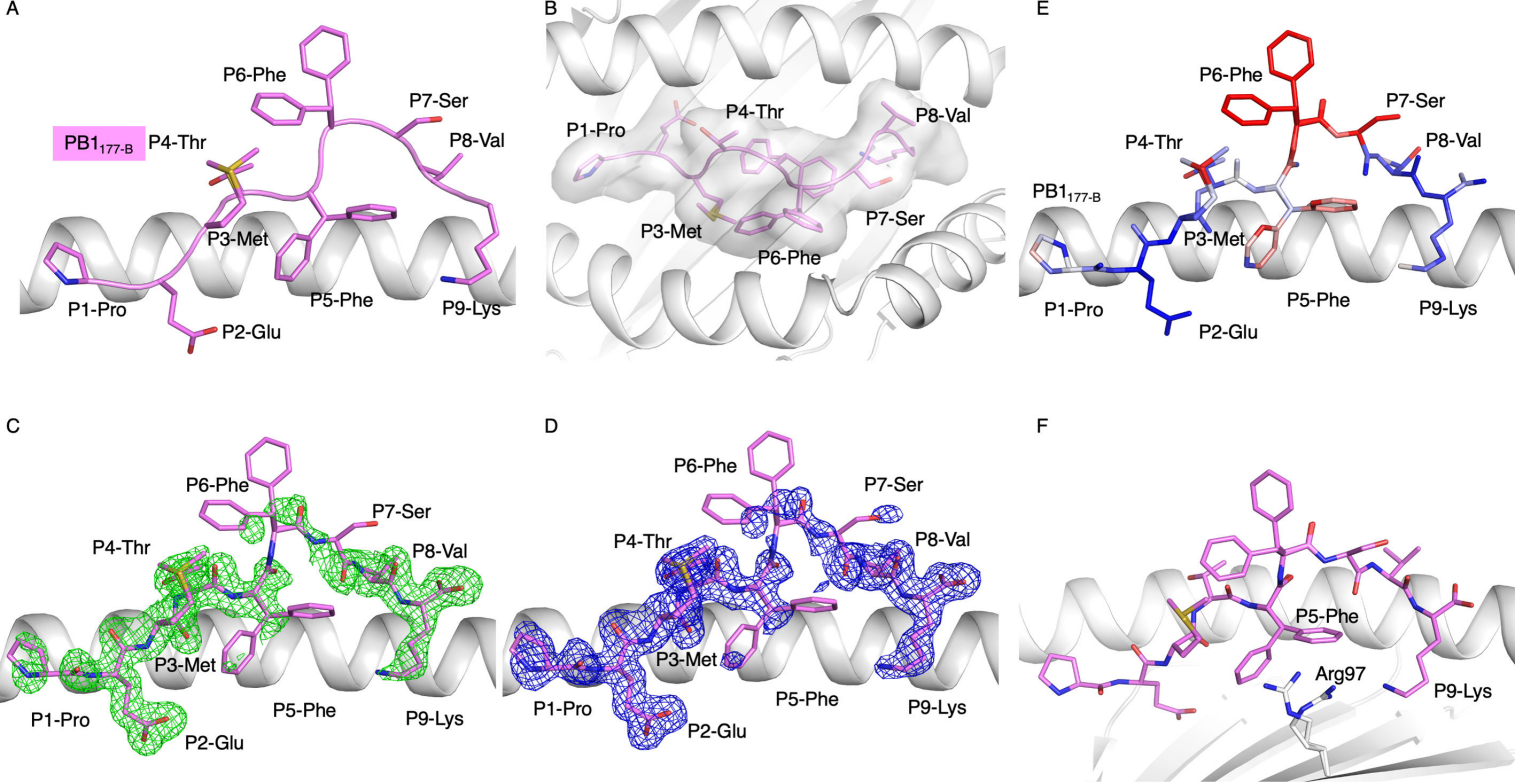
Crystal structure for HLA-B*18:01 presenting PB1_177-B_. (**A-B**) Side and top views, respectively, of PB1_177-B_ (pink cartoon and stick) presented by HLA-B*18:01 (white cartoon). The PB1_177-B_ peptide surface representation is added on panel (**B**). (**C-D**) Electron density representation of the (**C**) omit map (Fo-Fc at 3σ in green) and (**D**) the map after refinement (2Fo-Fc at 1σ in blue) of PB1_177-B_. (**E**) B-factor of PB1_177-B_ represented by a blue, white and red colour spectrum (20 Å² to 50 Å²). (**F**) HLA (white) and PB1_177-B_ (pink) interaction.

To understand the lack of CD8^+^ T cell activation observed in the samples tested for PB1_177-B_, we decided to solve the structure of the IBV-derived peptide to compare it with our previously solved IAV-derived PB1_177-A_ [16]. We first used AlphaFold2 [20] to generate a predicted model of HLA-B*18:01 presenting PB1_177-B_. The predicted structure showed binding of the PB1_177-B_ peptide where P8-Val constitutes the anchor residue in the F pocket instead of P9-Lys, which is the last residue (supplementary Figure 2). This anchor position causes the P9-Lys residue to be outside the antigen binding cleft. While it has been observed that the HLA-I molecule can present peptides with a C-terminal extension outside of the antigen binding cleft [21-25], a valine residue is an unfavourable residue within the F pocket of HLA-B*18:01, preferring larger residues at PΩ (Y/F/L/M/W) [17]. We then solved the X-ray crystal structure for the HLA-B*18:01-PB1_177-B_ complex at a resolution of 2.0 Å (Table 2). PB1_177-B_ adopts a canonical conformation within the binding cleft (Figure 2A), consistent with HLA-I binding [9]. This was in stark contrast with the AlphaFold2 predicted structure (supplementary Figure 2). The PB1_177-B_ side chains P2-Glu and P9-Lys are the primary anchors within the B and F pockets, respectively (Supplementary Table S1). P9-Lys at the PΩ anchor is non-canonical for HLA-B*18:01, with this molecule preferring large hydrophobic residues at this position, as per previously published structures [16].

**Table 2:** Crystallographic data collection and refinement statistics of HLA-B*18:01 presenting PB1_177-B._

Data collection statistics	HLA-B*18:01-PB1_177-B_
Space group	P 2_1_ 2_1_ 2_1_
Cell dimensions (a, b, c) (Å)	50.82, 81.42, 110.35
Resolution (Å)	46.16–2.00 (2.05–2.00)
Total number of observations	118,465 (9026)
Nb of unique observations	30,914(2310)
Multiplicity	3.8 (3.9)
Completeness (%)	98 (99.6)
I/σ1	7.5 (2.2)
Rpim^1^ (%)	6.8 (33.9)
CC_1/2_ (%)	99.3 (77.3)
**Refinement statistics**	
Rfactor^2^ (%)	19.57
Rfree^2^ (%)	24.76
R.m.s.d. deviations from ideality	
Bond lengths (Å)	0.007
Bond angles (°)	0.874
Ramachandran plot (%)	
Favoured	98.94
Allowed	1.06
Disallowed	0
PDB code	9DY8

1 Rpim = ∑hkl [1/(N − 1)]1/2 ∑i | Ihkl, i − < Ihkl > | / ∑hkl < Ihkl >.

2 Rfactor = ∑hkl | | Fo | - | Fc | | / ∑hkl | Fo | for all data except ≈ 5% which were used for Rfree calculation.

The crystal structure showed that P5-Phe was buried into the D pocket (Figure 2A, Supplementary Table S1). The P4-Thr, P6-Phe, P7-Ser and P8-Val side chains were solvent exposed, forming a large surface for potential TCR contacts (Figure 2B). Strikingly, there was high flexibility of the peptide central region, from P5-Phe to P7-Ser, as reflected by the weak electron density for the two residues (Figure 2C and D). This area had a higher B-factor compared with the rest of the peptide (Figure 2E), demonstrating the flexibility of the PB1_177-B_ peptide central region. P5-Phe facing down in the cleft may be inducing this flexibility due to the unfavourable interaction between this large hydrophobic residue and the positively charged Arg97 side chain at the base of the C pocket (Figure 2F).

### PB1_177-B_ flexibility destabilises the pHLA-B*18:01 complex

Given the unfavourable presence of the large aromatic P5-Phe residue in the HLA-B*18:01 C/D pockets (Supplementary Table S2), this could affect the overall stability of the pHLA-B*18:01 complex. To test this, a differential scattering fluorimetry assay was performed with the HLA-B*18:01-PB1_177-B_ complex (Supplementary Table S2) and compared with our previously published data for the HLA-B*18:01-PB1_177-A_ complex [16]. The melting temperature of the HLA-B*18:01-PB1_177-B_ complex was ~49°C (Table 1). This is considerably lower than the HLA-B*18:01-PB1_177-A_ complex (Tm~64°C), or even other peptide-HLA-B*18:01 complexes previously tested (Tm>60°C) [16]. While the overlay of both crystal structures of PB1_177-B_ and PB1_177-A_ presented by HLA-B*18:01 shows a similar backbone orientation (), the B-factor analysis showed that the central region of PB1_177-A_ was less flexible () and in turn has an overall higher *T*_m_ ().

**Figure 3:**
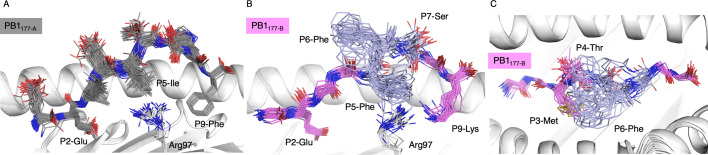
HLA-B*18:01 presenting peptides PB1_177-A_ and PB1_177-B_ ensemble refinement. Ensemble refinement side view of the HLA-B*18:01 cleft (white cartoon) with (**A**) PB1_177-B_ (grey sticks) and (**B**) of PB1_177-A_ (pink sticks). (**C**) Top view of the HLA-B*18:01 cleft with PB1_177-B_ (pink) flexible side chains (purple).

To understand the impact of the lower *T*_m_ on PB1_177-B_ peptide dynamics, we used a time-averaged ensemble refinement for both HLA-B*18:01-PB1_177-B_ and HLA-B*18:01-PB1_177-A_ (Figure 3). The ensemble models showed that Arg97 at the bottom of the C pocket exhibits some flexibility in both structures reflecting the unfavourable interaction between Arg97 and P5-Ile of PB1_177-A_ (Figure 3A) and P5-Phe of PB1_177-B_ (Figure 3B).

This is in contrast to the structure of HLA-B*18:01 presenting the IAV-derived M1_5_ peptide (A/X‐31 /H3N2), which has a similar melting temperature to PB1_177-A_ (Table 1) [16]. This peptide contains a small polar P5-Thr which was previously shown to form a hydrogen bond with Arg97 (Supplementary Figure 4C). In contrast to the PB1_177_ peptides, the M1_5_ B-factor analysis showed a rigid central peptide region (Supplementary Figure 4D). The ensemble model for M1_5_ presented by HLA-B*18:01 showed that Arg97 is flexible (Supplementary Figure 4E), suggesting that the lower *T*_m_ value observed for PB1_177-B_ peptide is due to the peptide flexibility and not the HLA.

As described above, the F pocket of HLA-B*18:01 favours hydrophobic residues like Phe/Ile. However, the IBV-derived peptide has a charged P9-Lys that is less favourable in the F pocket compared with large hydrophobic residues present at this position. This was exemplified in the ensemble refinement models, where the P9-Lys on the PB1_177-B_ could adopt different conformations (Figure 3B), while the PB1_177-A_ P9-Phe remained in the same orientation (Figure 3A). Altogether, this shows that the flexibility of the PB1_177-B_ peptide that led to lower thermal stability of the pHLA complex was due to less favourable primary and secondary anchor residues. The ensemble refinement for PB1_177-B_ also shows the large flexibility of P6-Phe, which can ‘shield’ P3-Met and P4-Thr, which could potentially limit TCR interaction (Figure 3C). This effect has previously been suggested to prevent TCR docking on HLA-B*18:01 presenting the NS2_111_ peptide (A/X‐31 /H3N2) due to a hydrogen bond with the HLA Glu155 [16].

Overall, the high flexibility of the PB1_177-B_ peptide observed might explain the lack of stimulation of CD8^+^ T cells, in contrast with the immunogenic PB1_177-A_ peptide.

## Discussion

The impact of the multiple lockdown restrictions during the COVID-19 pandemic has been suggested to reduce influenza virus circulation in the human population, which led to a decreased immunity and a predicted increased susceptibility by 10–60% towards IAV and IBV [26]. However, little is known about the immune response to IBV, and whether CD8^+^ T cells may provide cross-protection against IBV infection.

Therefore, we intended to provide a better understanding of HLA-I peptide presentation in the context of an IBV-derived peptide. Using a combination of cellular and molecular biological assays, we were able to show that despite binding to HLA-B*18:01, the PB1_177-B_ peptide was not immunogenic in the samples tested. A limitation of our study is that only three HLA-B*18:01^+^ samples were tested, and the previous infection and vaccination histories of these donors are unknown. However, it is of note that one of these samples was tested previously and showed CD8^+^ T cell activation towards the homologous IAV-derived PB1_177-A_ peptide [16]. Further, it is worth noting that the PB1_177-B_ peptide has not yet been reported to be naturally processed during influenza virus infection.

The HLA-B*18:01 preferred anchor residues are glutamic acid at P2 and a hydrophobic residue at PΩ [15,17,27,28]. Therefore, the presence of a charged PΩ-Lys in the PB1_177-B_ peptide is undesirable and might explain the lower stability of the HLA-B*18:01 in complex with the IBV-derived peptide, compared with the IAV-derived peptide. As previously shown in other crystal structures of both HLA-I [29] and HLA-II molecules [30], molecular dynamics can be used to highlight the role that residues within the centre of the peptide play in reducing the stability of the pHLA complex and overall CD8^+^ T cell activation. Here, we show that the PB1_177-B_ unfavourable P5-Phe secondary anchor’s flexibility affected the solvent-exposed P6-Phe that might in turn limit TCR recognition. The lower stability may affect pHLA complex trafficking to the cell surface or the half-life of the complex on the cell surface, which would in turn decrease the likelihood of T cell recognition. Therefore, while the PB1_177-A_ is immunogenic and stable in the HLA-B*18:01 cleft, its IBV-derived counterpart was neither and therefore might not be a good target for a cross-protective CD8^+^ T cell-based vaccine against both IAV and IBV.

Overall, this study uses biochemical techniques in combination with cellular immunology to characterise an IBV-derived peptide homologous to an IAV immunogenic peptide. We show that the IBV peptide was able to bind the HLA-B*18:01 molecule, despite uncanonical anchor residues, but not as stably as its IAV homologous peptide. We solved the first structure of an IBV-derived peptide presented by HLA-B*18:01 and showed the high dynamics of this peptide that might represent an obstacle for T cell recognition. This work provides a deeper understanding of the interplay between viral peptides and HLA interaction, which is vital for both TCR recognition and T cell activation.

## Methods

### Isolation of PBMCs

Whole peripheral blood from volunteer donors and buffy coats obtained from the Australian Red Cross Life Blood were processed and PBMCs were isolated using the Ficoll-gradient density method outlined in our previous publication [31]. All PBMCs were HLA typed (AlloSeq Tx) by CareDx Pty Ltd (Fremantle, Western Australia). Research was approved by the La Trobe University Human Ethics Committee (Approval No: HEC21097) and undertaken in accordance with the Declaration of Helsinki.

### PBMCs stimulation with the PB1_177-B_ peptide

The PB1_177-B_ (PEMTFFSVK) peptide was ordered at >80% purity from GenScript (Piscataway, U.S.A.). CD8^+^ T cell lines were generated from PBMCs of healthy HLA-B*18:01^+^ individuals stimulated with the PB1_177-B_ peptide as previously described [32]. One-third of PBMCs were peptide pulsed with 10 µM of reconstituted PB1_177-B_ peptide and were incubated at 37°C with 5% CO_2_ for 1.5 hr. PB1_177-B_ stimulated PBMCs were then washed twice and added to the remaining two-thirds of unstimulated PBMCs [32]. The PB1_177-B_-specific T cell lines were cultured for over 10 days in RPMI-1640 (Gibco, New York, U.S.A.) supplemented with 1 x Penicillin Streptomycin Glutamine (Gibco, New York, U.S.A.), 1 x non-essential amino acids (100 x NEAA) (Gibco, New York, U.S.A.), 5 mM HEPES (Sigma-Aldrich, St. Louis, U.S.A.), 2 mM L-glutamine (Sigma-Aldrich, St. Louis, U.S.A.), foetal bovine serum (FBS; Bovogen, Melbourne, Australia) and 50 µM of β-mercaptoethanol (Sigma-Aldrich, St. Louis, U.S.A.). On the fourth and seventh day, 10 IU/ml of recombinant human IL2 (Peprotech, Rocky Hill, U.S.A.) was added. 

### Intracellular cytokine staining (ICS) of PB1_177-B_-specific CD8^+^ T cell lines

PB1_177-B_-specific cell lines were counted using the trypan blue exclusion method to obtain 1.5 × 10^5^ cells/well for each cell line. PB1_177-B_-specific CD8^+^ T cell lines were then stimulated with either 10 µM of PB1_177-B_ peptide, Cell Stimulation Cocktail 500 x (phorbol 12-myristate 13-acetate (PMA) and ionomycin, eBiosciences, San Diego, U.S.A.) as a positive control, or nil peptide as a negative control. Cells were then incubated at 37°C with 5% CO_2_ for 5 hr with Golgi-stop (1:400; BD Biosciences, Franklin Lakes, U.S.A.), Golgi-Plug (1:1000; BD Biosciences, Franklin Lakes, U.S.A.) and anti-human CD107a-AF488 (1:200; eBiosciences, San Diego, U.S.A.). After 5 hr, cells were surface stained for 30 mins at 4°C with anti-human CD3-BV480 (1:100; BD Biosciences, Franklin Lakes, U.S.A.), anti-human CD4-BV650 (1:100; BD Biosciences, Franklin Lakes, U.S.A.), anti-human CD8-PerCPCy5.5 (1:50; BD Biosciences, Franklin Lakes, U.S.A.), anti-human CD14-APCH7 (1:100; BD Biosciences, Franklin Lakes, U.S.A.), anti-human CD19-APCH7 (1:100; BD Biosciences, Franklin Lakes, U.S.A.) and Live/Dead-NIR (1:1000; Molecular Probes, Eugene, U.S.A.). Following this, all cells were washed with PBS and then fixed and permeabilised with BD-Fix Perm buffer (BD Biosciences, Franklin Lakes, U.S.A.) at 4°C for 20 min. Finally, cells were intracellularly stained at 4°C for 30 min with anti-human IFNγ-BV421 (1:100; BD Biosciences, Franklin Lakes, U.S.A.), anti-human TNF-PeCy7 (1:100; BD Biosciences, Franklin Lakes, U.S.A.), anti-human MIP1β-APC (1:100; BD Biosciences, Franklin Lakes, U.S.A.) and anti-human IL2-PE (1:50; BD Biosciences, Franklin Lakes, U.S.A.) diluted in Perm-wash buffer (BD Biosciences, Franklin Lakes, U.S.A.). All cells were acquired on the BD FACSymphony A3 analyser (BD Biosciences, Franklin Lakes, U.S.A.) and analysed using FlowJo (BD Biosciences, Franklin Lakes, U.S.A.) version 10.10.0.

### Protein expression and purification

The sequence encoding HLA-B*18:01 heavy chain was sourced from the IPD-IMGT/HLA database [8]. To create a soluble protein construct lacking the transmembrane domain, the corresponding cDNA was sub-cloned into the pET30 plasmid vector using NedI/HindIII restriction enzymes (GenScript, Piscataway, U.S.A.). Both the recombinant protein and human β2-microglobulin (β2m) were individually expressed in BL21-RIL *Escherichia coli*, resulting in the expression of the protein in inclusion bodies that were subsequently extracted and purified [33]. Refolding of the protein involved using 5 mg of peptide, 30 mg of HLA-B*18:01 heavy chain and 10 mg of β2m in a refolding buffer comprising 3 M urea (ThermoFisher, Melbourne, Australia), 0.4 M L-arginine (Merck, Darmstadt, Germany), 0.1 M Tris-HCl pH 8.0 (ThermoFisher, Melbourne, Australia), 2 mM Na-EDTA pH 8.0 (VWR chemicals, Leuven, Belgium), 0.16% w/v reduced glutathione (Goldbio, St Louis, U.S.A.), and 0.03% w/v oxidised glutathione (Goldbio, St Louis, U.S.A.). Following refolding, the solution was dialysed within 10 mM Tris-HCl pH 8.0 (ThermoFisher, Melbourne, Australia), and the peptide-HLA*-*B*18:01 complexes were purified using two-stage anion exchange chromatography (Cytiva, Marlborough, U.S.A.).

### Crystallisation, structure determination and refinement process

Crystallisation of HLA-B*18:01-PB1_177-B_ complex at 6 mg/ml in a solution of 10  mM Tris-HCl pH 8.0 (ThermoFisher, Scoresby, Australia) and 150  mM NaCl (ThermoFisher, Melbourne, Australia) was achieved in sitting drop through vapour diffusion at 20°C. Crystals for HLA-B*18:01-PB1_177-B_ grew in 0.2 M Lithium Acetate and 20% polyethylene glycol 3350. Crystals were then soaked into cryoprotection solution containing the mother liquor and 20% Ethylene glycol (Hampton Research, Aliso Viejo, U.S.A.) and flash frozen in liquid nitrogen. Diffraction data set was collected on the MX2 beamline at the ANSTO Australian Synchrotron and processed using XDS [34]. Structure was solved by molecular replacement software PHASER v.2.8.3 [35] via the CCP4 software suit v8.0.005 [36] with a HLA-B*18:01 structure as model with the peptide removed (PDB code 6MT3 [15]). COOT v0.9.8.4 [37] was used to build the model, followed by PHENIX v1.20-1-4487-000 [38] for both refinement and ensemble refinement, with both programmes set at default parameters. The PDB OnDep System was then used to validate and deposit the final model under the PDB accession number 9DY8. The AlphaFold2 model was built using the Google Colab integrated ChimeraX AlphaFold tool [39]. All figure representations were generated with PyMoL (v2.5).

### Differential scanning fluorimetry

The ViiA 7 real-time PCR system (ThermoFisher, Melbourne, Australia) was used to run the thermal stability assay for the HLA-B*18:01-PB1_177-B_ complex. TAMRA reporter (x3−m3 filter) was used with an excitation wavelength of approximately 550  nm and emission detection around 587  nm. Protein complexes were diluted to concentrations of 5 and 10 μM in 10  mM Tris-HCl pH 8.0 and 150  mM NaCl, with duplicate samples at each concentration. SYPRO Orange dye (ThermoFisher, Melbourne, Australia) was then added to achieve a final concentration of 10 x. Samples were heated from 25°C to 95°C at a rate of 1 °C/min with emission readings taken at each 0.5°C. The fluorescence data was normalised using GraphPad Prism 9 (v9.3), and the thermal melting point (Tm) of each complex was determined as the temperature corresponding to 50% of the maximum fluorescence intensity. This assay was conducted with two independent samples in duplicate at each concentration.*

## Supplementary material

Online supplementary material

## Data Availability

The data generated and/or analysed during the study are available from the corresponding author upon reasonable request. The data supporting the findings of this study are included within the article and its supplementary materials. The X-ray crystallography structure is available on the PDB website under the accession number 9DY8.

## Abbreviations

HA: hemagglutinin
HLA: human leukocyte antigen
IAV: influenza A virus
IBV: influenza B virus
NA: neuraminidase
TCR: T cell receptor

## References

[1] Iuliano A.D. Roguski K.M. Chang H.H. Muscatello D.J. Palekar R. Tempia S. et al. Estimates of global seasonal influenza-associated respiratory mortality: a modelling study Lancet 391 10127 10.1016/S0140-6736(17)33293-2 PMC5935243 29248255

[2] Muraduzzaman A.K.M. Illing P.T. Mifsud N.A. Purcell A.W 2022 Understanding the role of HLA class I molecules in the immune response to influenza infection and rational design of a peptide-based vaccine Viruses 14 2578 10.3390/v14112578 36423187 PMC9695287

[3] Nuwarda R.F. Alharbi A.A. Kayser V 2021 An overview of Influenza viruses and vaccines Vaccines (Basel) 9 1032 10.3390/vaccines9091032 34579269 PMC8473132

[4] Gouglas D. Thanh Le T. Henderson K. Kaloudis A. Danielsen T. Hammersland N.C. et al. 2018 Estimating the cost of vaccine development against epidemic infectious diseases: a cost minimisation study Lancet Glob. Health 6 e1386 e1396 10.1016/S2214-109X(18)30346-2 30342925 PMC7164811

[5] Eiden J. Volckaert B. Rudenko O. Aitchison R. Herber R. Belshe R. et al. 2022 M2-deficient single-replication Influenza vaccine-induced immune responses associated with protection against human challenge with highly drifted H3N2 Influenza strain J. Infect. Dis. 226 83 90 10.1093/infdis/jiab374 34323977 PMC9373152

[6] van de Sandt C.E. Dou Y. Vogelzang-van Trierum S.E. Westgeest K.B. Pronk M.R. Osterhaus A.D.M.E. et al. 2015 Influenza B virus-specific CD8+ T-lymphocytes strongly cross-react with viruses of the opposing influenza B lineage J. Gen. Virol. 96 2061 2073 10.1099/vir.0.000156 25900135 PMC4681061

[7] Ljunggren H.G. Stam N.J. Öhlén C. Neefjes J.J. Höglund P. Heemels M.T. et al. 1990 Empty MHC class I molecules come out in the cold Nature 346 476 480 10.1038/346476a0 2198471

[8] Barker D.J. Maccari G. Georgiou X. Cooper M.A. Flicek P. Robinson J. et al. 2023 The IPD-IMGT/HLA database Nucleic Acids Res. 51 D1053 D1060 10.1093/nar/gkac1011 36350643 PMC9825470

[9] Nguyen A.T. Szeto C. Gras S 2021 The pockets guide to HLA class I molecules Biochem. Soc. Trans. 49 2319 2331 10.1042/BST20210410 34581761 PMC8589423

[10] Leong S.L. Gras S. Grant E.J 2024 Fighting flu: novel CD8+ T-cell targets are required for future influenza vaccines Clin. Transl. Immunology 13 e1491 10.1002/cti2.1491 38362528 PMC10867544

[11] Zaraket H. Hurt A.C. Clinch B. Barr I. Lee N Lee N.(2021) burden of influenza B virus infection and considerations for clinical management Antiviral Res. 104970 10.1016/j.antiviral.2020.104970 33159999

[12] Tran D. Vaudry W. Moore D. Bettinger J.A. Halperin S.A. Scheifele D.W. et al. 2016 Hospitalization for Influenza A Versus B Pediatrics 138 2015 4643 10.1542/peds.2015-4643 27535144

[13] Liu C.Y. Wang J.D. Yu J.T. Wang L.C. Lin M.C. Lee H.F. et al. 2014 Influenza B virus-associated pneumonia in pediatric patients: clinical features, laboratory data, and chest X-ray findings Pediatr. Neonatol. 55 58 64 10.1016/j.pedneo.2013.07.002 24113227

[14] Faviel F. McCabe A. Santos Eduardo J.M.D. Jones J. Takeshita L. Ortega-Rivera Nestor D 2019 Allele frequency net database (AFND) 2020 update: gold-standard data classification, open access genotype data and new query tools Nucleic Acids Res. 48 D783 D788 10.1093/nar/gkz1029 PMC7145554 31722398

[15] Grant E.J. Josephs T.M. Loh L. Clemens E.B. Sant S. Bharadwaj M. et al. 2018 Broad CD8^+^ T cell cross-recognition of distinct influenza A strains in humans Nat. Commun. 9 5427 10.1038/s41467-018-07815-5 30575715 PMC6303473

[16] Leong S.L. Murdolo L. Maddumage J.C. Koutsakos M. Kedzierska K. Purcell A.W. et al. 2024 Characterisation of novel influenza-derived HLA-B*18:01-restricted epitopes Clin. Transl. Immunology 13 e1509 10.1002/cti2.1509 38737448 PMC11087170

[17] Jurtz V. Paul S. Andreatta M. Marcatili P. Peters B. Nielsen M 2017 NetMHCpan-4.0: improved peptide-MHC class I interaction predictions integrating eluted ligand and peptide binding affinity data J. Immunol. 199 3360 3368 10.4049/jimmunol.1700893 28978689 PMC5679736

[18] Madeira F. Madhusoodanan N. Lee J. Eusebi A. Niewielska A. Tivey A.R.N. et al. 2024 The EMBL-EBI Job Dispatcher sequence analysis tools framework in 2024 Nucleic Acids Res. 52 W521 W525 10.1093/nar/gkae241 38597606 PMC11223882

[19] McCluskey J. Gras S.J. Bharadwaj M. Kjer-Nielsen L. Macdonald W.A. Saunders P.M. et al. 2010 Mehra N.K. edHLA Molecules of the Major Histocompatibility Complex1st ededIndia DK Agencies (P) Ltd

[20] Jumper J. Evans R. Pritzel A. Green T. Figurnov M. Ronneberger O. et al. 2021 Highly accurate protein structure prediction with AlphaFold Nature 596 583 589 10.1038/s41586-021-03819-2 34265844 PMC8371605

[21] Collins E.J. Garboczi D.N. Wiley D.C 1994 Three-dimensional structure of a peptide extending from one end of a class I MHC binding site Nature 371 626 629 10.1038/371626a0 7935798

[22] Tenzer S. Wee E. Burgevin A. Stewart-Jones G. Friis L. Lamberth K. et al. 2009 Antigen processing influences HIV-specific cytotoxic T lymphocyte immunodominance Nat. Immunol. 10 636 646 10.1038/ni.1728 19412183

[23] Remesh S.G. Andreatta M. Ying G. Kaever T. Nielsen M. McMurtrey C. et al. 2017 Unconventional peptide presentation by major histocompatibility complex (MHC) class I Allele HLA-A*02:01: BREAKING CONFINEMENT J. Biol. Chem. 292 5262 5270 10.1074/jbc.M117.776542 28179428 PMC5392673

[24] Guillaume P. Picaud S. Baumgaertner P. Montandon N. Schmidt J. Speiser D.E. et al. 2018 The C-terminal extension landscape of naturally presented HLA-I ligands Proc. Natl. Acad. Sci. U.S.A. 115 5083 5088 10.1073/pnas.1717277115 29712860 PMC5960288

[25] Hensen L. Illing P.T. Bridie Clemens E. Nguyen T.H.O. Koutsakos M. van de Sandt C.E. et al. 2021 CD8+ T cell landscape in Indigenous and non-Indigenous people restricted by influenza mortality-associated HLA-A*24:02 allomorph Nat. Commun. 12 2931 10.1038/s41467-021-23212-x 34006841 PMC8132304

[26] Ali S.T. Lau Y.C. Shan S. Ryu S. Du Z. Wang L. et al. 2022 Prediction of upcoming global infection burden of influenza seasons after relaxation of public health and social measures during the COVID-19 pandemic: a modelling study Lancet Glob. Health 10 e1612 e1622 10.1016/S2214-109X(22)00358-8 36240828 PMC9573849

[27] Rist M.J. Theodossis A. Croft N.P. Neller M.A. Welland A. Chen Z. et al. 2013 HLA peptide length preferences control CD8+ T cell responses The Journal of Immunology 191 561 571 10.4049/jimmunol.1300292 23749632

[28] Rist M.J. Hibbert K.M. Croft N.P. Smith C. Neller M.A. Burrows J.M. et al. 2015 T cell cross-reactivity between a highly immunogenic EBV epitope and a self-peptide naturally presented by HLA-B*18:01+ cells J. Immunol. 194 4668 4675 10.4049/jimmunol.1500233 25855358

[29] Fodor J. Riley B.T. Borg N.A. Buckle A.M 2018 Previously hidden dynamics at the TCR-peptide-MHC interface revealed J. Immunol. 200 4134 4145 10.4049/jimmunol.1800315 29728507

[30] Szeto C. Bloom J.I. Sloane H. Lobos C.A. Fodor J. Jayasinghe D. et al. 2020 Impact of HLA-DR antigen binding cleft rigidity on T cell recognition Int. J. Mol. Sci. 21 7081 10.3390/ijms21197081 32992915 PMC7582474

[31] Lineburg K.E. Grant E.J. Swaminathan S. Chatzileontiadou D.S.M. Szeto C. Sloane H. et al. 2021 CD8^+^ T cells specific for an immunodominant SARS-CoV-2 nucleocapsid epitope cross-react with selective seasonal coronaviruses Immunity 54 1055 1065 10.1016/j.immuni.2021.04.006 33945786 PMC8043652

[32] Grant E.J. Gras S 2022 Protocol for generation of human peptide-specific primary CD8+ T cell lines STAR Protocols 3 101590 10.1016/j.xpro.2022.101590 35942343 PMC9356162

[33] Chatzileontiadou D.S.M. Szeto C. Jayasinghe D Protein purification and crystallization of HLA-A*02:01 in complex with SARS-CoV-2 peptides STAR Protocols 2 100635 10.1016/j.xpro.2021.100635 PMC8188458 34124695

[34] Kabsch W 2010 Acta crystallogr D biol crystallogr XDS 66 125 132 10.1107/s0907444909047337 PMC2815665 20124692

[35] McCoy A.J. Grosse-Kunstleve R.W. Adams P.D. Winn M.D. Storoni L.C. Read R 2007 Phaser crystallographic software J Appl Crystallogr 40 658 674 10.1107/S0021889807021206 19461840 PMC2483472

[36] Agirre J. Atanasova M. Bagdonas H. Ballard C.B. Baslé A. Beilsten-Edmands J. et al. 2023 The CCP4 suite: integrative software for macromolecular crystallography Acta Crystallogr. D. Struct. Biol. 79 449 461 10.1107/S2059798323003595 37259835 PMC10233625

[37] Emsley P. Lohkamp B. Scott W.G. Cowtan K 2010 Features and development of Coot Acta Crystallogr. D Biol. Crystallogr. 66 486 501 https://doi.org/10.1107.S0907444910007493 20383002 10.1107/S0907444910007493 PMC2852313

[38] Liebschner D. Afonine P.V. Baker M.L. Bunkóczi G. Chen V.B. Croll T.I. et al. 2019 Macromolecular structure determination using X-rays, neutrons and electrons: recent developments in Phenix Acta Crystallogr. D. Struct. Biol. 75 861 877 10.1107/S2059798319011471 31588918 PMC6778852

[39] Varadi M. Anyango S. Deshpande M. Nair S. Natassia C. Yordanova G. et al. 2022 AlphaFold protein structure database: massively expanding the structural coverage of protein-sequence space with high-accuracy models Nucleic Acids Res. 50 D439 D444 10.1093/nar/gkab1061 34791371 PMC8728224

