# Supplementary material for: Characterisation of an influenza B virus-derived peptide presented by HLA-B*18:01
Source: Biochem J. 2025 Jul 16;482(14):811–20. doi: 10.1042/BCJ20240739 (PMC12409988; doi:10.1042/BCJ20240739)
Supplement: Online supplementary material [file bcj-482-14-BCJ20240739-s001.docx]

**Supplementary Data**

**Characterisation of an Influenza B virus-derived peptide**

**presented by HLA-B*18:01**

Lawton D. Murdolo^1,2*^, Samuel Liwei Leong^1,2*^, Janesha C. Maddumage^1,2^, Nicole A Mifsud^3^, Demetra S.M. Chatzileontiadou^1,2,4^, Emma J. Grant^1,2,4#^, Stephanie Gras^1,2,4#^

**Supplementary Table 1. Residues forming the peptide binding pocket of HLA-B*18:01**

| **Pocket** | **HLA-B*18:01 residue** |
| --- | --- |
| A | Met5, Tyr7, Try59, Asn63, Thr163, Trp167, His171 |
| B | His9, Ser24, Ile66, Ser67 |
| C | Thr69, Asn70, Thr73, Typ74, Arg97 |
| D | Tyr99, Tyr159 |
| E | Asp114, Trp147, Ala150, Val152, Gln155, Leu156 |
| F | Glu76, Ser77, Asn80, Try84, Leu95, Ser116, Tyr123, Thr143, Lys146 |

**Supplementary Table 2. List of HLA-B*18:01 contacts with PB1_177-B_**

| Peptide residue | HLA residue |
| --- | --- |
| P1-Pro | Tyr7, Tyr59, Tyr159, Try167 |
| P2-Glu^N-Oe1^ | Tyr7, His9^Nε2^, Asp63^Oδ1^, Ile66 |
| P3-Met^N-O^ | Asp70^Nδ2^, Tyr99^OH^, Leu156, Tyr159 |
| P4-Thr | Ile66, Thr69 |
| P5-Phe^N^ | His8, Asp70^Oδ1^, Thr73, Tyr74, Tyr99, Trp147, Leu156 |
| P6-Phe | Aln150, Glu155, Leu156 |
| P7-Ser | Thr73, Trp147 |
| P8-Val | Thr73, Gly76, Ser77 |
| P9-Lys^N-OXT^ | Ser77^Oδ^, Asp80^Nδ2^, Tyr84, Leu95, Ser116, Tyr123, Thr143, Lys146, Trp147 |

Hydrogen bond cut off ≤ 3.5Å, Van der Walls contacts cut off **≤** 4Å.

**
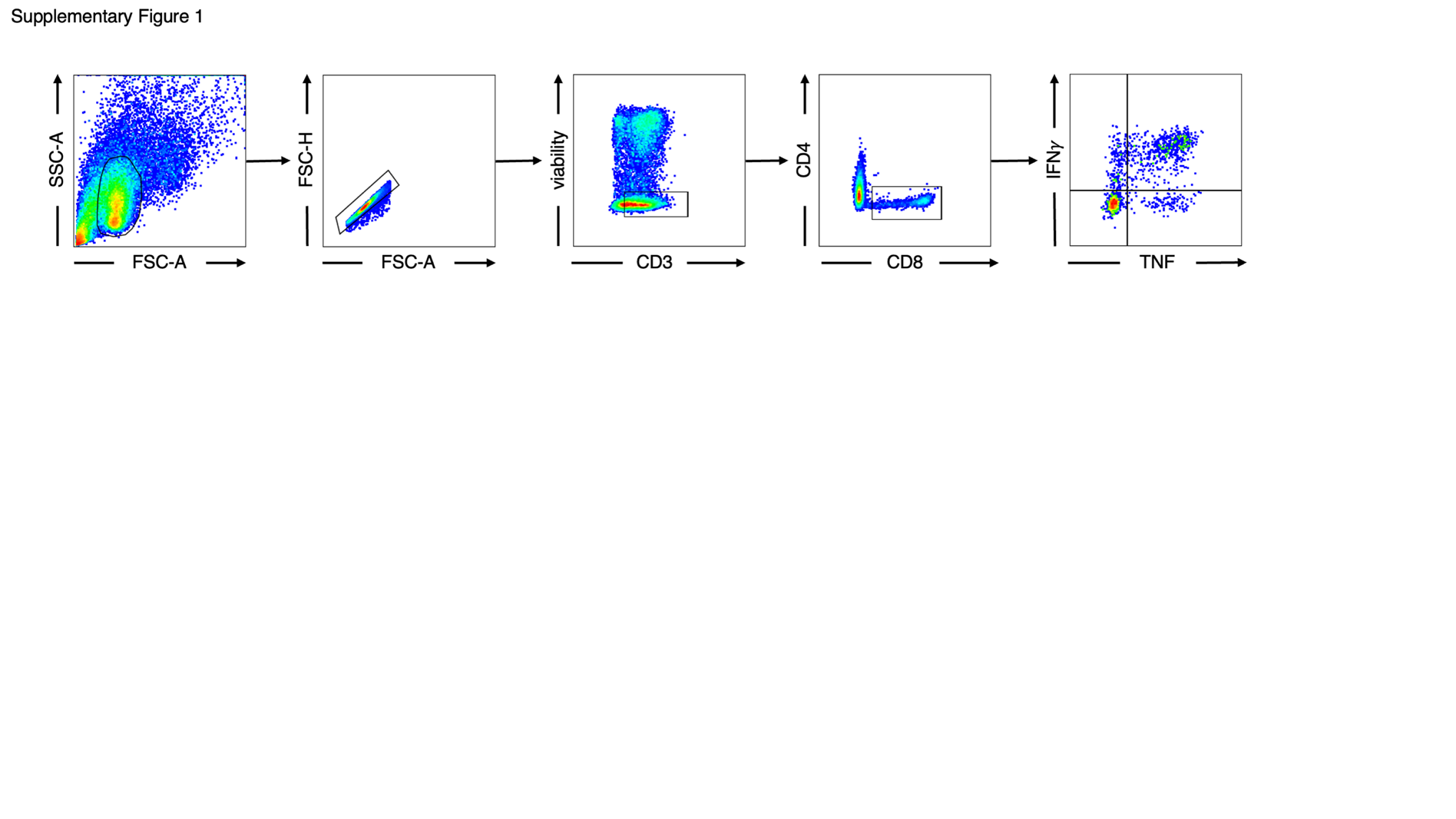
**

**Supplementary Figure 1. Gating strategy**

Representative FACS plots of a CD8^+^ T cell line stimulated with the 500x positive control, demonstrating the gating strategy used to assess CD8^+^ T cell responses in an ICS assay. Cells were gated on lymphocytes, singlets, LiveCD3^mid-high^, CD4^-^CD8^mid-high^ T cells and IFNγ and TNF were assessed as a marker of activation.

**
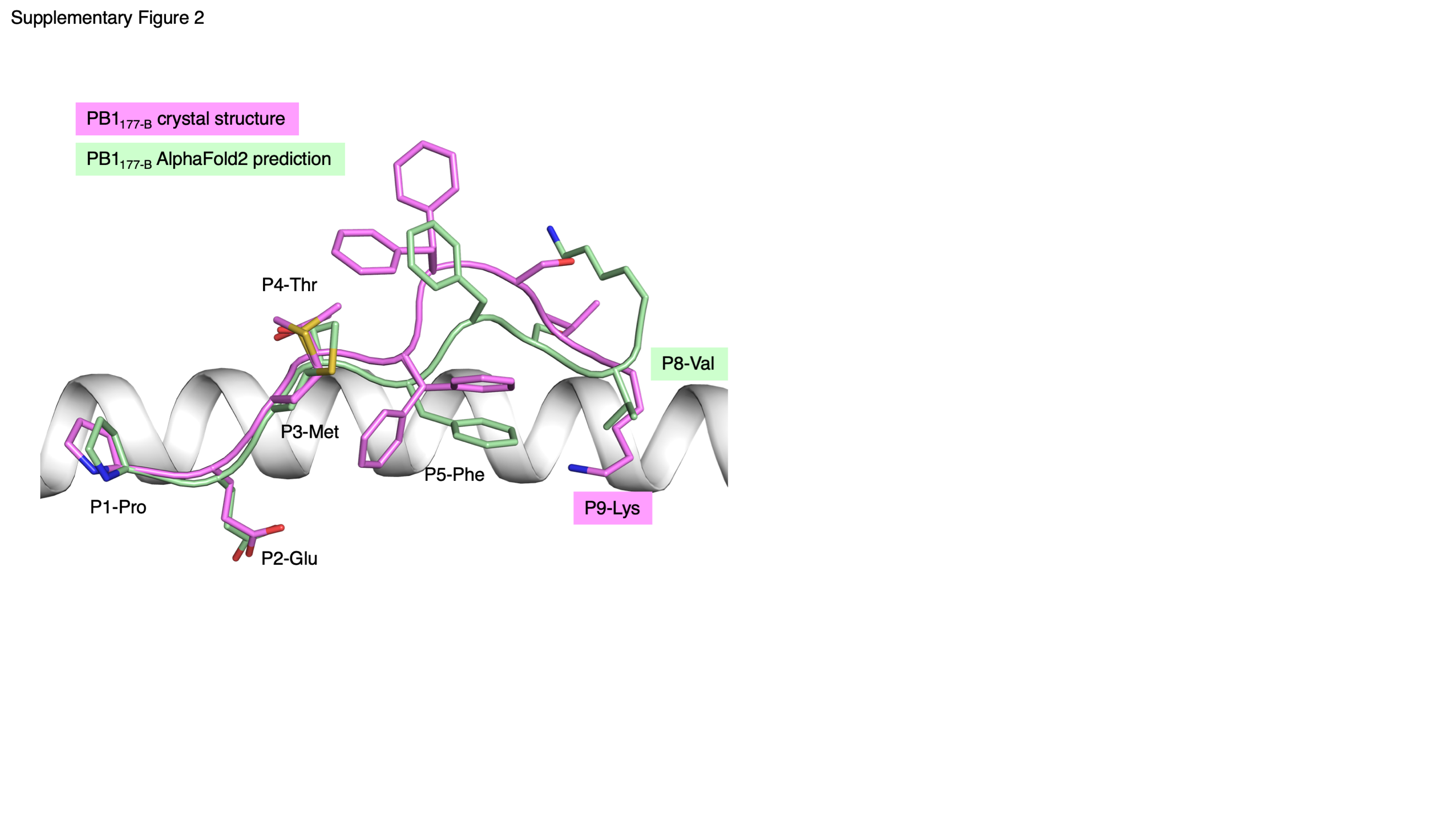
**

**Supplementary Figure 2. AlphaFold2 model of HLA-B*18:01 presenting PB1_177-B_**

Overlay of the AlphaFold2 structure (green sticks) and the experimental structure determined by X-ray crystallography (pink sticks) with each peptide’s PΩ labelled. The HLA-B*18:01 helix is represented as white cartoon.

**
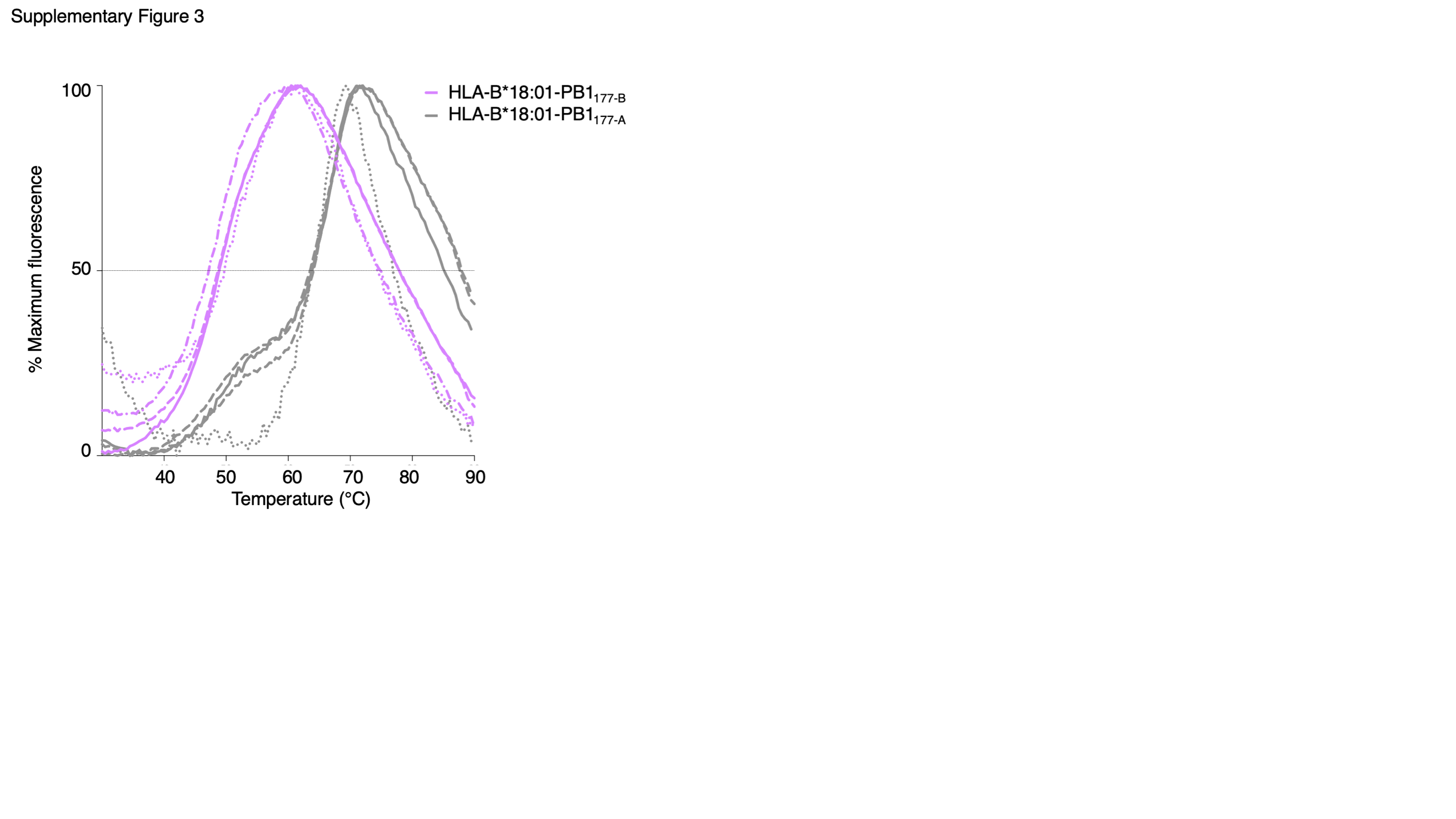
**

**Supplementary Figure 3. Thermal stability of HLA-B*18:01 presenting PB1_177-B_**

The DSF normalized florescence intensity (%) plotted against temperature (°C) at two protein concentrations of 5μM and 10μM. Two biologically independent experiments (n=2) were performed with each duplicate shown as different lines. The pink lines represent the thermal melt curves for the HLA-B*18:01-PB1_177-B_ and the grey for HLA-B*18:01-PB1_177-A_.

**
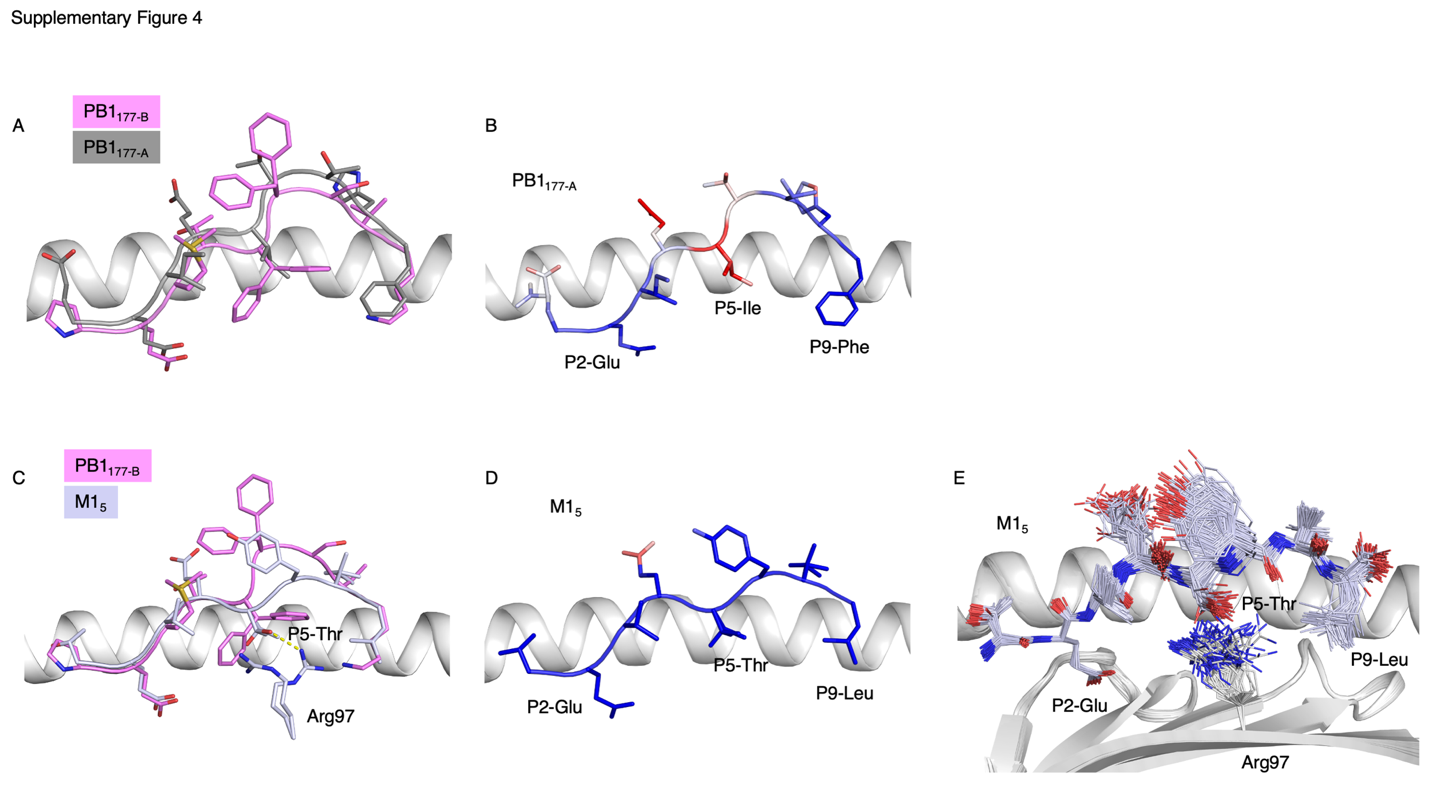
Supplementary Figure 4. HLA-B*18:01 presenting PB1_117-A_ and M1_5_**

Overlay of HLA-B*18:01 presenting PB1_177-B_ (pink cartoon) with (A) PB1_177-A_ (grey cartoon) and (C) M1_5_ (light blue cartoon). B-factor of (B) PB1_117-A_ and (D) M1_5_ represented by a blue, white and red color spectrum (20Å² to 50Å², respectively). Ensemble refinement of (E) M1_5_ with backbone and side chain (blue sticks, respectively) within the HLA-B*18:01 cleft (white cartoon).
